# Circulating Angiopoietin-like proteins in *Strongyloides Stercoralis* infection and reversal following treatment

**DOI:** 10.1371/journal.pntd.0013559

**Published:** 2025-09-30

**Authors:** Anuradha Rajamanickam, Saravanan Munisankar, Subash Babu

**Affiliations:** 1 National Institute of Health -International Center for Excellence in Research, Chennai, India; 2 Laboratory of Parasitic Diseases, National Institute of Allergy and Infectious Diseases, National Institutes of Health, Bethesda, Maryland, United States of America; George Washington University School of Medicine and Health Sciences, UNITED STATES OF AMERICA

## Abstract

*Strongyloides stercoralis (Ss)* is a parasitic infection affecting 50–100 million people globally, with significant immune and metabolic consequences, particularly in immunocompromised individuals. While Strongyloides infection is known to modulate the host immune system, the role of angiopoietin-like proteins (AGPTLs), which regulate inflammation and immune responses, has not been explored in this context. In this study, we investigated the systemic levels of AGPTL-2, -3, -4, -6, and -8 in 60 Ss-infected (*Ss*+) and 56 uninfected (*Ss*-) individuals. AGPTL levels were quantified using enzyme-linked immunosorbent assays (ELISA). We also assessed the effect of anthelmintic treatment on AGPTL levels in *Ss*+ individuals. Our results show that *Ss+* individuals had significantly elevated levels of AGPTL-2, -3, -6, and -8 compared to Ss- individuals. After anthelmintic treatment, these elevated levels were significantly reduced. Principal component analysis (PCA) revealed distinct clustering of AGPTLs between *Ss*+ and *Ss*- groups, explaining 25.1% and 36.1% of the variance, respectively. Additionally, a positive correlation between AGPTL levels and IgG suggested an association with immune activation. These findings suggest that Strongyloides infection is associated with elevated AGPTL levels, which decrease following effective treatment. This highlights the potential role of AGPTLs as biomarkers for diagnosing and monitoring infection and treatment response. Further research is needed to better understand the mechanisms of AGPTL regulation in parasitic infections and their impact on immune modulation and metabolic alterations.

## Author summary

*Strongyloides stercoralis* is a parasitic worm that infects people mostly in tropical and subtropical regions, often without causing noticeable symptoms. However, in individuals with weakened immune systems, the infection can become severe and even life-threatening. Our study examined at a group of proteins in the body called angiopoietin-like proteins (AGPTLs), which help regulate inflammation and immune responses. These proteins had not been previously studied in people with Strongyloides infection. We compared the levels of AGPTLs in individuals with and without the infection and found that four of these proteins (AGPTL-2, 3, 6, and 8) were significantly higher in those infected. Following anthelmintic treatment, the levels of these proteins decreased. We also observed that higher levels of these proteins were linked to immune responses in the body, suggesting they may play a role in how the body responds to the parasite. Our results suggest that AGPTLs could be useful markers for identifying infection and tracking how well treatment works. They may also help us understand how parasitic infections affect the immune system and possibly protect against other conditions like diabetes.

## Introduction

Strongyloidiasis is a soil-transmitted disease and *Strongyloides stercoralis* (*Ss*) is the causative agent. *Ss* infection affects an estimated 50–100 million people worldwide, with increased infection rates in tropical and subtropical areas where favorable environmental conditions, inadequate sanitation, and poor hygiene [1–3]. *Ss* exhibits a unique reproductive ability, with an auto-infective cycle in the human host and sexual reproduction in the soil [4]. *Ss* infection is often clinically asymptomatic for years or even decades and can modulate the host immune system due to its chronic nature. However, in immunocompromised patients, uncontrolled parasite multiplication (hyper-infection) and the potentially life-threatening dissemination of larvae can lead to mortality rates of up to 85% [5,6]. Chronic Strongyloides infection can cause cutaneous, gastrointestinal, and/or pulmonary symptoms, and in immunosuppressed individuals, it may progress to hyper-infection syndrome or disseminated strongyloidiasis, both of which can be potentially fatal [5,7]. Both innate and adaptive immune responses contribute to the defense against *Ss* infection. The immune response is primarily Th2-dominated, characterized by elevated levels of Th2 cytokines, including IL-4, IL-5, and IL-13, as well as eosinophilia, which help regulate the parasite infection. However, *S. stercoralis* employs immunomodulatory mechanisms that allow the parasite to evade immune detection, contributing to its chronic persistence in the host [8].

Angiopoietin-like proteins (ANGPTLs) are secreted glycoproteins structurally similar to angiopoietins, involved in processes such as angiogenesis, lipid metabolism, and inflammation [9]. Key members, including ANGPTL 2, 3, 4, 6, and 8, regulate angiogenesis and vascular permeability, contributing to tissue remodeling during infections [10]. ANGPTL 2, in particular, plays a significant role in immune regulation and inflammatory responses and has been associated with various inflammatory diseases, including cardiovascular conditions and chronic infections [9]. However, the role of ANGPTLs in *S. stercoralis* infection remains underexplored.

We hypothesized that ANGPTLs may be involved in modulating the host’s immune response and influencing the chronicity of the infection. *Ss* infection, known for its ability to persist in the host for years, elicits a complex immune response. Understanding how ANGPTLs regulate immune responses during *Ss* infection could provide new insights into the pathophysiology of the disease and offer potential targets for therapeutic intervention.

## Materials and methods

### Ethics statement

All individuals were examined as part of a natural history study protocol approved by the Institutional Review Boards of the National Institute of Allergy and Infectious Diseases (USA) and the National Institute for Research in Tuberculosis (India) (12-I-073), and informed written consent was obtained from all participants.

### Study population

We studied a total of 116 individuals comprising 60 clinically asymptomatic, Strongyloides-infected (here, *Ss*+) individuals and 56 uninfected, healthy (here, *Ss*-) individuals in Tamil Nadu, South India. All *Ss*+ individuals were confirmed by stool agar plate culture and serology, and because the participants were asymptomatic, they all represented chronic infections. There were no instances of acute strongyloidiasis included.

In order to minimize confounding factors, exclusion criteria included HIV infection, other intestinal helminths (by stool microscopy), prior anti-helminthic treatment, and diagnosed metabolic or inflammatory disorders. These individuals were all recruited from a rural population by screening individuals for helminth infection by stool microscopy and serology. Inclusion criteria were the age of 18–65 years and willingness to give blood and stool samples for examination; exclusion criteria were past antihelminth treatment, other helminth infections, or HIV infection. Follow-up was performed at 6 months following recruitment and treatment. Strongyloides infection was diagnosed by the presence of IgG antibodies to the recombinant antigen, NIE, as described previously [1,11]. This was further confirmed by specialized stool examination with nutrient agar plate cultures [12]. None of the study population had lymphatic filariasis or other intestinal helminths (based on stool microscopy). All infected individuals were treated with single doses of ivermectin and albendazole, and follow-up blood draws were obtained 6 months later. All participants had negative stool cultures at follow-up, confirming the cure. As anticipated, at six months, Strongyloides IgG decreased but did not completely return to seronegative in every individual. All uninfected individuals were anti-Strongyloides NIE negative and negative for filarial and other intestinal helminths.

### Hematological parameters

Leukocyte counts and differentials were performed on all individuals using an AcT 5 Diff haematology analyzer (Beckman Coulter).

### Measurement of Angiopoietin-like proteins

Systemic levels of Angiopoietin-like proteins (ANGPTL) 3, 4, and 6 were determined using the R&D systems Duoset ELISA kit method. The lower detection limits were as follows: ANGPTL-3: 31.2 pg/mL; ANGPTL -4: 1.3 ng/mL; ANGPTL -6: 78.1 pg/mL respectively. Angiopoietin-like proteins (ANGPTL) 2 and 8 were determined using the MyBiosources ELISA kit method. The lower detection limits were as follows: ANGPTL - 2: 0.312 ng/mL and ANGPTL - 8: 125 pg/mL.

### Statistical analysis

Data analyses were performed using GraphPad Prism 10.6.0 (GraphPad Software, Inc., San Diego, CA, USA). Geometric means (GM) were used for measurements of central tendencies. Comparisons were made using either a Mann-Whitney U test for comparisons between two groups or a Wilcoxon signed-rank test for comparisons within groups. Corrections for multiple comparisons were performed by Holm’s correction.

## Results

### Study population characteristics

The baseline demographic characteristics and hematological and biochemical parameters have been described in Table 1. There were no significant differences in age, gender, and biochemical parameters between the two groups. In contrast, BMI (Body Mass Index) (median of *Ss* + 19.5 Kg/m^2^ Vs. *Ss*- 22.7 Kg/m^2^, p = 0.0001), RBC (Red Blood Cells) (median of *Ss* + 4.1 *10*^*6*^*/ml* Vs. *Ss*- 4.7 *10*^*6*^*/ml,* p = 0.0246), and Hb (Hemoglobin) (median of *Ss* + 12.05 g/dL Vs. *Ss*- 13.5 g/dL, p = 0.0003) levels were significantly lower in the *Ss*+ group in comparison with the *Ss*- group. On the other hand, the percentage of eosinophils (median of *Ss* + 11% Vs. *Ss*- 6.2%, p = 0.0001) and basophils (median of *Ss* + 1% Vs. *Ss*- 0.8%, p = 0.0023) levels were significantly higher in the *Ss*+ group in comparison with the *Ss*- group.

**Table 1:** Demographics, haematological and biochemical parameters of the study population.

*Parameters*	*Ss+ (n = 60)*	*Ss- (n = 56)*	*p value*
*Gender (Male/Female)*	29/31	24/32	0.7212
*Median age in Years(range)*	44 (18 - 64)	37 (20 - 63)	0.1986
*BMI* Kg/m^2^ *(range)*	19.5 (18.6-24.4)	22.7 (19.6 - 24.6)	0.0001
*WBC count, x10* ^*9*^ */L*	8.5 (3.7 - 12.2)	8.5 (5.3 -13.8)	0.4316
*Lymphocyte %*	27.9 (10.8 - 39.2)	28.5 (1.35 - 42.7)	0.8161
*Neutrophil %*	51.5 (33.10 - 72.20)	53.6 (3.03 - 73.10)	0.7381
*Monocyte %*	6.8 (3.8 - 13.7)	7.1 (33.1 - 711.6)	0.7312
*Eosinophil %*	11 (1.1 - 34.5)	6.2 (0.19 – 29.2)	0.0001
*Basophil %*	1 (0.4 - 25.8)	0.8 (0.04 – 3.5)	0.0023
*RBC, x10* ^*6*^ */ml*	4.1 (2.11 - 5.84)	4.7 (3.5 -12.6)	0.0246
*Hb, g/dL*	12.05 (6.9 – 16.10)	13.5 (8.7 - 18.50)	0.0003
*Hematocrit, %*	39.5 (21.7 – 54.8)	37.25 (15 - 49)	0.1565
*Platelet, 10* ^*3*^ */uL*	266 (165 - 455)	273 (160 - 427)	0.7906
***Biochemical Parameters***			
* Random Blood Glucose (RBG) mg/dl*	99 (60 - 168)	101 (63 - 186)	0.2131
* HbA1c %*	5.9 (4.5 – 7.9)	6.07 (4.7 - 12)	0.0781
* AST U/L*	21 (13 - 79)	25 (14 - 90)	0.0816
* ALT U/L*	19 (7 - 25)	22 (7 – 48)	0.1117
* Urea mg/dl*	21 (11 - 35)	26 (10 - 40)	0.2101
* Creatinine mg/dl*	0.7 (0.4 – 1.2)	0.8 (0.4 1.2)	0.4436

### Post-treatment follow-up

All *Ss*+ individuals had negative stool cultures six months after treatment, indicating a parasitological cure. Even though not everyone became seronegative at this point, IgG levels against the NIE antigen decreased (S1 Table).

### Strongyloides infection is associated with increased levels of angiopoietin-like proteins

**Fig 1:**
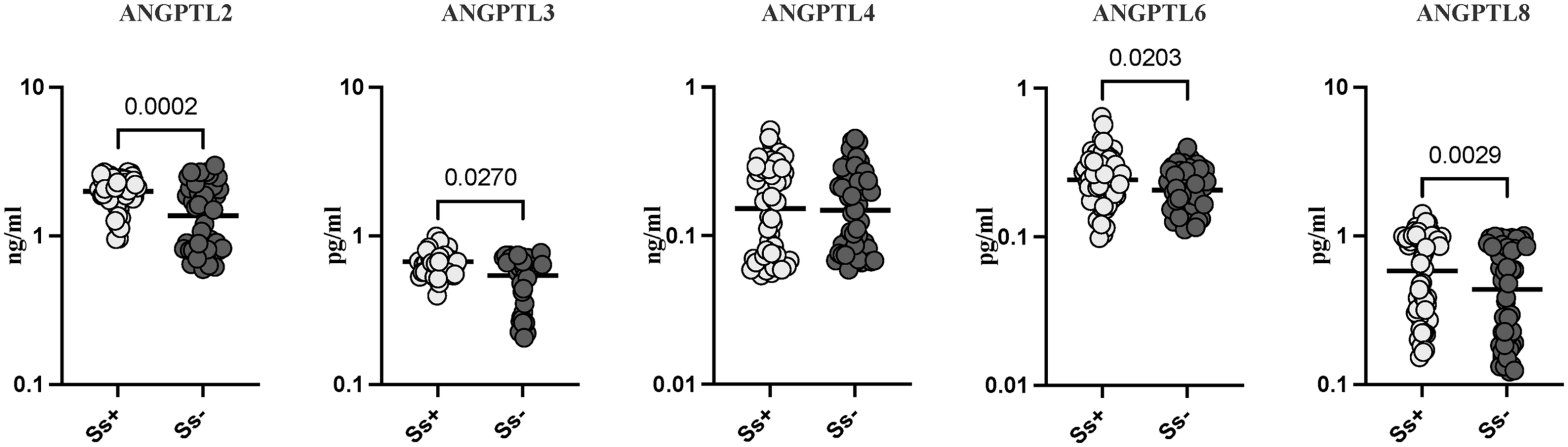
Strongyloides infection is associated with increased levels of angiopoietin-like proteins. (A) Plasma levels of Angiopoietin-like proteins (AGPTLs) AGPTL-2, 3, 4, 6, and 8 in *Ss* infected (*Ss* + n = 60)and *Ss* uninfected (*Ss*- (n = 56) individuals. Each dot is an individual subject with the bar representing the geometric mean (GM). Mann– Whitney U-test with Holms correction for multiple comparisons were done by p-values are multiplied by the number of parameters.

To determine the systemic levels of Angiopoietin-like proteins (AGPTLs) profiles in Strongyloides infection, we measured the circulating levels of AGPTL-2, 3, 4, 6, and 8 in *Ss* infected and *Ss* uninfected individuals. As shown in Fig 1, the systemic levels of the AGPTL-2 (GM of 1.99 pg/ml in *Ss+* versus 1.37 pg/ml in *Ss-*; p = 0.0002), AGPTL-3 (GM of 0.67 pg/ml in *Ss+* versus 0.54 pg/ml in *Ss-*; p = 0.0270), AGPTL-6 (GM of 0.24 pg/ml in *Ss+* versus 0.21 pg/ml in *Ss-*; p = 0.0203) and AGPTL-8 (GM of 0.58 pg/ml in *Ss+* versus 0.43 pg/ml in *Ss-*; p = 0.0029) were significantly increased in *Ss* infected individuals compared with *Ss*- individuals. Thus, *Ss* infection is associated with increased levels of angiopoietin-like proteins.

### Alterations in systemic levels of angiopoietin-like proteins following treatment of Strongyloides infection

**Fig 2:**
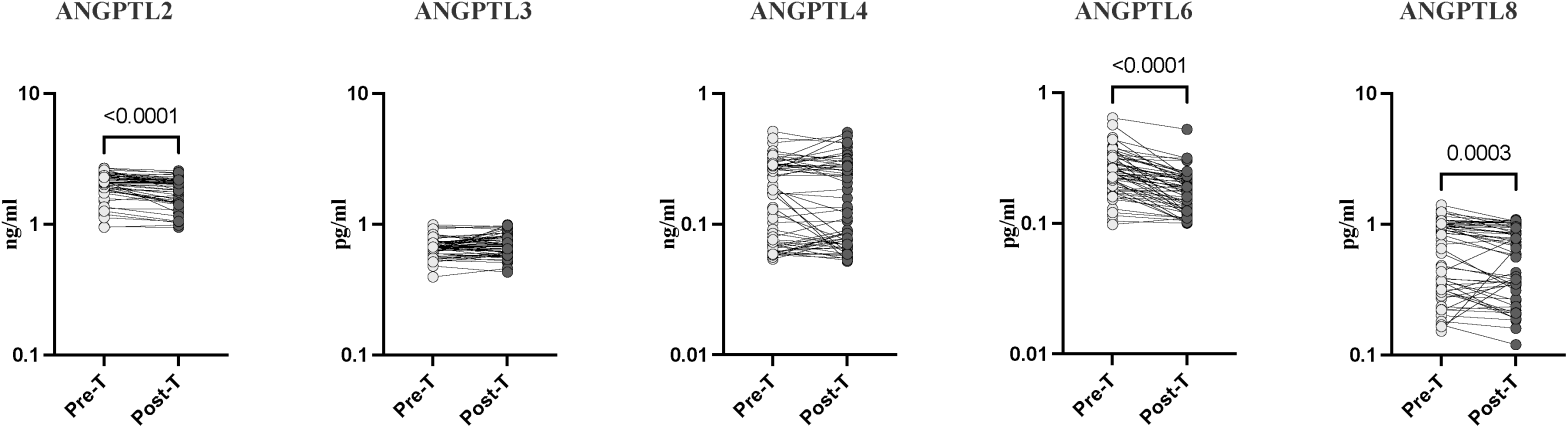
Reversal of systemic levels of angiopoietin-like proteins following treatment of Strongyloides infection. (A) Plasma levels of Angiopoietin-like proteins (AGPTLs) AGPTL-2, 3, 4, 6, and 8 in *Ss* infected *Ss* + pre-treatment [Pre-T] [n = 60] and 6 months following treatment [post-T] [n = 60] were measured. were measured. p values were calculated using the Wilcoxon matched pair test.

To determine the effect of treatment on the systemic Angiopoietin-like proteins (AGPTL) profile in Strongyloides infection, we measured the circulating levels of AGPTL 2, 3, 4, 6, and 8 in *Ss* infected individuals before and after treatment. As shown in Fig 2, the post-treated individuals had significantly decreased levels of AGPTL-2 (fold change 1.1 pg/ml; p < 0.0001); AGPTL-6 (fold change 1.4 pg/ml; p < 0.0001); and AGPTL-6 (fold change 1.1 pg/ml; p = 0.0003) compared to pre-treated individuals. Thus, anthelmintic therapy significantly reverses the levels of angiopoietin-like proteins in *Ss*+ individuals.

### Multivariate regression analysis of angiopoietin-like proteins

Multivariate regression analysis was performed to evaluate the impact of *Ss* infection on the diverse parameters measured in this study. As indicated in Table 2, after adjusting for the influence of age and sex, AGPTL-2, 3, 6, and 8 were all significantly impacted by *Ss* infection. Thus, our data confirm that *Ss* infection profoundly influences various important parameters, including levels of angiopoietin-like proteins.

**Table 2:** Factors associated with the *SS* Positives.

Factors	OR (95% CI)	p Value	aOR^a^ (95% CI)	p Value
Age	1.25 (0.63 - 3.22)	0.622		
Gender				
* *Female	1.00			
* *Male	0.85 (0.70-1.03)	0.09		
* *IgG AU/L	10.6 (4.19 - 33.65)	0.001	8.6 (2.69 - 28.23)	0.001
* *BMI Kg/m^2^	1.20 (1.05-1.37)	0.005	1.18 (1.03-1.35)	0.004
* *HbA1c %	0.79 (0.21 - 2.94)	0.722	0.89 (0.24 - 3.39)	0.868
* *RBG mg/dl	1.01 (0.98 - 1.03)	0.514	1.01 (0.98 - 1.04)	0.525
* *Urea mg/dl	1.05 (0.95 - 1.16)	0.318	1.05 (0.94 - 1.17)	0.377
* *Creatinine mg/dl	0.07 (0.00 - 1.78)	0.107	0.03 (0.00 - 1.31)	0.070
* *ALT U/L	1.00 (0.97 - 1.04)	0.877	1.04 (0.99 - 1.10)	0.145
* *AST U/L	0.98 (0.94 - 1.02)	0.346	0.96 (0.90 - 1.02)	0.162
* *AGPTL2	1.36 (0.39-4.77)	0.633	1.06 (0.24-4.65)	0.036
* *AGPTL3	0.86 (0.61 - 0.94)	0.015	0.72 (0.61 - 0.94)	0.015
* *AGPTL4	1.11 (0.58 - 2.11)	0.759	1.10 (0.58 - 2.09)	0.772
* *AGPTL6	0.68 (0.52 - 0.89)	0.003	0.68 (0.52 - 0.89)	0.003
* *AGPTL8	3.2 (1.68 - 5.76)	0.005	3.21 (1.68 - 5.27)	0.008

^a^Adjusted Odds Ratio for age and gender; OR – Odds Ratio.

### Principle component analysis reveals trends in angiopoietin-like proteins

**Fig 3:**
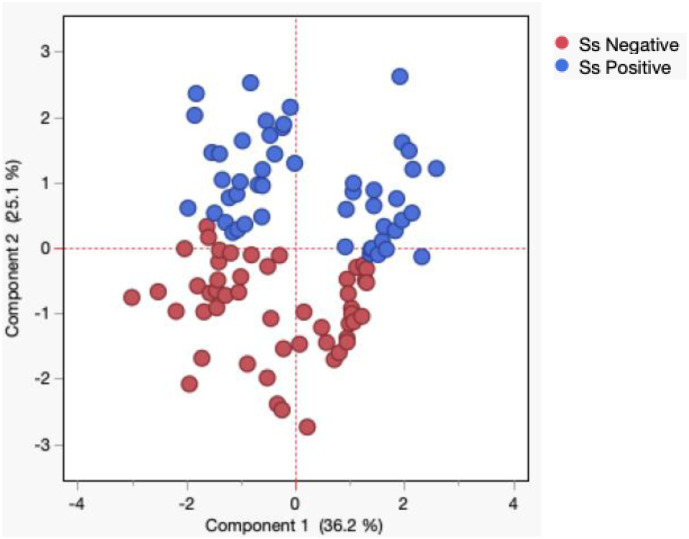
Principle component analysis reveals trends in angiopoietin-like proteins. Principal component analysis (PCA) was performed to show the distribution of data from the combination of three groups *Ss*+ (blue circles), and *Ss*- (maroon circle). The PCA represents the two principal components of variation. PCA analysis was performed with angiopoietin-like proteins between *Ss*+ and *Ss*- individuals.

PCA was used to visualize differences between the groups created on the entire data set. We performed PCA with angiopoietin-like proteins (AGPTL-2, 3, 4, 6, and 8) to visualize the clustering pattern of angiopoietin-like proteins in individuals with and without *Ss* infection. After excluding those factors with commonalities as low as 0.5, we assessed PCA-1 (AGPTL-2, 3, 4) and PCA-2 (AGPTL-6, and 8). As illustrated in Fig 3, PCA analysis showed that angiopoietin-like protein clusters varied between *Ss*+ and *Ss*- individuals. The score plot of the first two components revealed 25.1% and 36.2% of overall variance, respectively.

### Relationship between IgG and angiopoietin-like proteins

To examine the relationship between IgG and angiopoietin-like proteins, we assessed the association of these parameters with IgG in all the individuals in the study. As shown in Table 3, the systemic levels of AGPTL-2, 6, and 8 exhibited a significant positive association with the levels of IgG. Thus, IgG levels against *Ss* were positively correlated with the circulating levels of angiopoietin-like proteins.

**Table 3:** Correlation (Spearman’s) between AGPTL levels and IgG. A multiparametric matrix correlation plot depicts the relationships between plasma AGPTL-2, 3, 4, 6, and 8 and IgG levels in *Ss* infected (*Ss* + n = 60) and *Ss* uninfected (*Ss*- (n = 56) individuals.

Variables	Spearman’s p	p-value
AGPTL-2 vs IgG	0.7143	<0.001
AGPTL-3 vs IgG	0.15	0.0520
AGPTL-4 vs IgG	0.09	0.6541
AGPTL-6 vs IgG	0.36	0.0110
AGPTL-8 vs IgG	0.48	0.0302

## Discussion

The results of this study provide valuable insights into the systemic changes that occur during *Ss* infection, specifically with regard to the levels of angiopoietin-like proteins (AGPTLs). Our findings show that *Ss* infection is associated with significant changes in both hematological and biochemical parameters, as well as modulation in the systemic circulating levels of AGPTLs. In addition, following anthelmintic therapy, the AGPTLs levels were significantly reversed in *Ss*-infected individuals.

Our study revealed significant differences in hematological parameters between *Ss*+ (infected) and *Ss*- (uninfected) individuals. We observed lower levels of body mass index (BMI), red blood cells (RBCs), and hemoglobin (Hb) in the *Ss*+ group. The lower BMI in the Ss+ group suggests potential malnutrition or altered metabolism, possibly due to malabsorption or chronic inflammation caused by the parasite. These findings are consistent with previous studies showing that Strongyloides infection can lead to nutritional deficiencies and anemia, possibly due to the increased metabolic demands of the parasite and its effects on the host’s gastrointestinal tract. The reduction in Hb and RBC levels may also be indicative of a chronic inflammatory response and immune dysregulation caused by the persistent infection [13–17]. Eosinophils, known for their pro-inflammatory and anti-inflammatory properties, are believed to play a crucial role in the innate immune response during parasitic infections [18,19]. The elevated eosinophil and basophil percentages in the *Ss*+ group reflect an ongoing immune response typical of *Strongyloides* infection, with eosinophilia being a hallmark of parasitic infestations. These results suggest a possible role in tissue remodeling, an immune response typical of parasitic infections, where these cells are involved in the inflammatory and allergic reactions induced by the infection [17,18,20–22].

Our study highlights a significant alteration in the systemic levels of angiopoietin-like proteins (AGPTLs) in individuals infected with *Ss*, suggesting a profound impact of this parasitic infection on the host’s immune responses. Specifically, circulating levels of AGPTL-2, AGPTL-3, AGPTL-6, and AGPTL-8 were significantly elevated in *Ss*+ compared to *Ss*- individuals. These proteins are known regulators of inflammation, immune modulation, angiogenesis, and lipid metabolism [23–25]. Previous studies have found that circulating ANGPTL-8 levels are elevated in inflammation-related diseases, such as systemic inflammatory response syndrome (SIRS) [26], T2DM [27], atherosclerosis [26], and NAFLD [9], are associated with disease severity. ANGPTL-8, a factor involved in regulating lipid and glucose metabolism, serves as a potential biomarker for metabolic diseases. It was also reported to be involved in the pathophysiological processes of circulatory system-related diseases as an inflammatory factor [28]. Notably, previous studies have indicated that helminth infections, such as those caused by *Strongyloides*, may have protective effects against the development or progression of type 2 diabetes mellitus (T2DM) [29–32]. Thus, while the primary role of these proteins is in immune modulation and vascular function [33–35], their increased levels during *Ss* infection may contribute to an indirect protective effect by supporting metabolic processes that may prevent or delay the onset of T2DM. These findings suggest that the modulation of AGPTLs in response to *Ss* infection could be one of the mechanisms through which helminth infections provide a protective effect against metabolic disorders like T2DM.

The increase in AGPTLs, particularly AGPTL-2 and AGPTL-6, observed in this study may reflect an ongoing inflammatory response, as these proteins are involved in immune regulation and vascular remodeling [35–37]. Elevated AGPTL levels could provide insights into the host’s immune response to *Strongyloides*. The interplay between these immune-regulatory proteins and metabolic processes could be a crucial factor in the observed protective effects of helminth infections on T2DM. For instance, ANGPTL-2 regulates vascular permeability and tissue remodeling, while ANGPTLs -3, -6, and -8 are involved in lipid metabolism and insulin sensitivity. Recent findings suggest that ANGPTL-2 is a key mediator linking obesity to systemic insulin resistance, playing a crucial role in both atherosclerosis and the development of diabetes. It also contributes to chronic endothelial and vascular inflammation, which promotes atherosclerosis [25,36,38]. ANGPTL-3 levels are elevated in patients with rheumatic disorders like dermatomyositis and systemic sclerosis. It is also linked to atherosclerosis and the levels of ANGPTL-3 and their relationship with lipid and glucose metabolic markers in patients recently diagnosed with T2DM [24,34,35,38,39]. Serum ANGPTL-6 levels are significantly higher in patients with metabolic syndrome, particularly in those with increased waist circumference or low HDL cholesterol. These findings appear to contradict earlier evidence suggesting that ANGPTL-6 helps counteract obesity and insulin resistance, warranting further research [38]. ANGPTL-8 is a liver-derived circulating factor that controls plasma triglyceride levels and is regarded as a crucial mediator in the post-meal movement of fatty acids to adipose tissue [24,34,38]. The elevated levels of these AGPTLs may indicate an adaptive immune response to control the infection but also reflect alterations in the host’s metabolic pathways that could help delay or prevent the onset of T2DM. These findings underscore the complex role of *Ss* infection in modulating immune responses, suggesting a potential mechanism through which helminth infections may have a role in delaying or preventing metabolic related disease like T2DM.

Furthermore, the study demonstrates that anthelmintic treatment significantly reduces the elevated levels of AGPTLs in *Ss*+ individuals, with AGPTL-2, AGPTL-6, and AGPTL-8 levels showing marked decreases post-treatment. This suggests that the elevated production of AGPTLs is closely linked to the presence of the helminth and can be reversed following effective treatment, underscoring the dynamic relationship between infection and immune modulation. Multivariate regression analysis confirmed that *Ss* infection significantly influences AGPTL levels, even after adjusting for age and sex. Additionally, principal component analysis (PCA) revealed distinct clustering patterns of AGPTLs between *Ss*+ and Ss- individuals, further supporting the notion that these proteins are indicative of *Ss* infection. Notably, a positive correlation was found between IgG levels and AGPTLs, suggesting that *Ss* infection induces both humoral and systemic immune responses, with higher IgG levels being associated with elevated AGPTLs. These findings show that IgG and AGPTLs are linked to the immune response in helminth infections. Collectively, our study highlights AGPTLs have a role in *Ss* infection immune responses.

This study highlights that *Ss* infection is associated with elevated levels of AGPTLs, specifically AGPTL-2, 3, 6, and 8, suggesting their role in the host’s immune response induced by the helminth. The anthelmintic treatment effectively reduced these elevated levels, supporting the potential use of AGPTLs as biomarkers for diagnosing and monitoring *Strongyloides* infection and evaluating treatment efficacy. However, the study has limitations, limited exploration of other AGPTL family members or immune markers, the need for long-term studies to assess the impact of treatment on AGPTL levels and clinical outcomes, and the lack of parasite-specific cellular immune responses. However, in order to determine how AGPTLs contribute to immune modulation and metabolic outcomes in the context of *Ss* infection, mechanistic studies will be crucial. Although known metabolic or inflammatory diseases, HIV, and other intestinal helminths were not included in the design, we recognize that AGPTL levels may be impacted by unidentified or subclinical comorbidities. This is a limitation that we have added. Since this comparison was cross-sectional, our results show correlations between AGPTL levels and infection status but are unable to prove causation. To define the biological role of AGPTLs in *Ss* infection, we emphasize that mechanistic studies are warranted. Furthermore, we observe that there was only one 6-month follow-up. This is in line with research on standard helminth treatment; however, multi-point, longer-term follow-up studies will be required to ascertain whether AGPTL. Although known metabolic or inflammatory diseases, HIV, and other intestinal helminths were not included in the design, we recognize that AGPTL levels may be impacted by unidentified or subclinical comorbidities. Our study opens new avenues for further research on helminth infections and ANGPTLs. The findings highlight the potential role of these proteins at the intersection of helminth infections, making them promising targets for therapeutic interventions in both helminth-related and metabolic syndrome treatments. Future research exploring the relationship between ANGPTLs and helminth infections, the broader role of AGPTLs in helminth infections, their mechanisms, and expansion to larger and more diverse populations of helminth infections, would provide valuable insights into new therapeutic approaches for managing both infectious and metabolic diseases.

## Supporting information

S1 Table Strongyloides stool culture and IgG results pre- and post-treatment. **(6 months)** NIE-specific IgG ELISA was used to measure the serological response, and nutrient agar plate stool culture was used to evaluate parasitological cure. Every *Ss*+ person had a stool culture that was positive at baseline and negative six months after treatment. In line with the slower kinetics of antibody responses, IgG levels significantly decreased following treatment, though not all patients returned to seronegativity within six months. Throughout, *Ss*− people stayed negative.
(DOCX)
