# Supplementary material for: Circulating Angiopoietin-like proteins in Strongyloides Stercoralis infection and reversal following treatment
Source: PLoS Negl Trop Dis. 2025 Sep 30;19(9):e0013559. doi: 10.1371/journal.pntd.0013559 (PMC12483219; doi:10.1371/journal.pntd.0013559)
Supplement: S1 Table — (6 months) NIE-specific IgG ELISA was used to measure the serological response, and nutrient agar plate stool culture was used to evaluate parasitological cure. Every Ss+ person had a stool culture that was positive at baseline and negative six months after treatment. In line with the slower kinetics of antibody responses, IgG levels significantly decreased following treatment, though not all patients returned to seronegativity within six months. Throughout, Ss− people stayed negative. (DOCX) [file pntd.0013559.s001.docx]

**Table S1. Strongyloides stool culture and IgG results pre- and post-treatment (6 months).**

| Participant group | n | Stool culture pre-T | Stool culture post-T | NIE IgG pre-T (median, IQR) | NIE IgG post-T (median, IQR) |
| --- | --- | --- | --- | --- | --- |
| Ss+ | 60 | 100% positive | 0% positive | 45 (30–58) | 32 (18–45) |
| Ss- | 56 | 0% positive | 0% positive | 5 (3–8) | 5 (3–7) |
